# Patient-reported outcome was close to the Danish background population 6 months after non-surgical treatment of Neer 2-part surgical neck fractures: a prospective cohort study in patients aged 60 or above

**DOI:** 10.2340/17453674.2024.42301

**Published:** 2024-11-05

**Authors:** Stig BRORSON, Signe A BORG, Line L HOUKJÆR, Kenneth B HOLTZ, Zaid ISSA

**Affiliations:** 1 Centre for Evidence-Based Orthopaedics, Department of Orthopaedic Surgery, Zealand University Hospital, Køge; 2 Department of Clinical Medicine, University of Copenhagen, Denmark

## Abstract

**Background and purpose:**

Neer 2-part surgical neck fractures are the most common displaced proximal humerus fractures. We aimed to evaluate patient-reported outcome in a consecutive series of older people receiving nonoperative treatment.

**Methods:**

This is a single-center prospective cohort study. We included patients aged 60 or above referred to a Danish university hospital. The preregistered protocol followed the recommendations from randomized trials. Patients were followed at the outpatient clinic at 2, 6, and 24 weeks. After 24 weeks, they were evaluated with Oxford Shoulder Score (OSS, 0–48, 48 best) and EuroQoL 5 dimensions, 3 levels (EQ-5D-3L, –0.624 to 1, 1 best). Clinical failure was defined as conversion to surgery or OSS ≤ 24. Population norms were reported to interpret the cohort data, but no formal statistical comparisons between historical cohorts were planned. We used descriptive statistics to report rates and proportions.

**Results:**

For 36 months, 268 patients (mean age 76, 79% female) with Neer 2-part surgical neck fractures received non-surgical treatment. After excluding patients with concomitant fractures, dementia, or death, complete follow-up was available for 167 patients. 8 patients (3.0%) had surgery. The mean OSS was 37.2 (SD 8.1), which equals 78% of maximum shoulder function. The norm for the population of the same age and gender was 82%. The mean EQ-5D-3L score was 0.79 (SD 0.16), while the norm for the same-age population was 0.82. 16 (10%) had an OSS score of 24 or below.

**Conclusion:**

Non-surgical treatment in older people with Neer 2-part surgical neck fractures resulted after 6 months in patient-reported shoulder function and quality of life close to that of the Danish background population.

More than half of proximal humerus fractures are displaced [1,2] of which the Neer 2-part surgical neck fracture is the most common, representing 28%. Females represent 76% of all cases [3]. The fracture often results in the humeral shaft being translated medially and anteriorly (Figure 1). This pattern has a distinct AO/OTA classification subgroup (AO A3.2), accounting for 13% of all proximal humerus fractures [2]. In some osteoporotic Neer 2-part fractures, the humeral head collapses into varus (AO subgroup A2.2), accounting for 13% (Figure 2).

**Figure 1:**
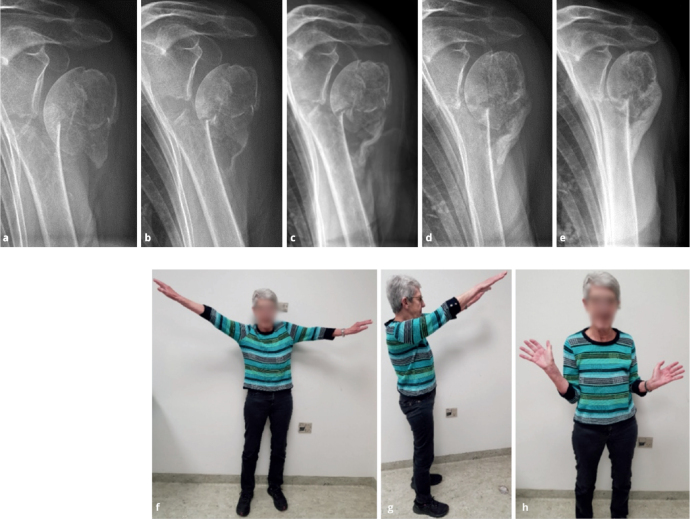
Left 2-part surgical neck fracture with medial translation of the humeral shaft in an 80-year-old female. Anterior–posterior radiographs at 2, 4, 6, 12, and 24 weeks (a–e). Photos (f–h) at 6 months after injury. At 24 weeks, OSS was 41 (84%), and EQ-5D-3L was 0.78.

**Figure 2:**
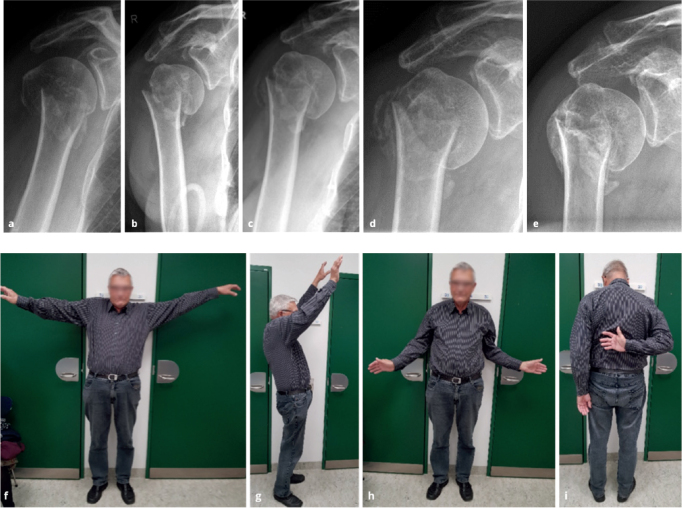
Right 2-part surgical neck fracture in a 73-year-old male. Progressive varus collapse of the humeral head occurred during the first 6 weeks. Solid healing and pain-free function at shoulder level were obtained. Anterior–posterior radiographs at admission, 1, 3, 6, 12, and 24 weeks (a–e). Photos 24 weeks after injury (f–i). At 24 weeks, OSS was 42 (86%), and EQ-5D-3L was 0.78

For decades, it has been believed that displaced Neer 2-part fractures needed open reduction and internal fixation. This practice has received support from commercial stakeholders and has been passed down to younger surgeons [4-7]. Recent randomized trials have failed to demonstrate the superiority of surgical interventions, which is why it is suggested that surgery for Neer 2-part fractures may not benefit older people [8]. However, there is limited data on patient-reported outcome after non-surgical treatments outside control groups in randomized trials.

We aimed to study patient-reported outcome 6 months after non-surgical treatment of Neer 2-part surgical neck fractures in a consecutive cohort of patients aged 60 or above compared with the background population.

## Methods

### Study design

**Figure 3:**
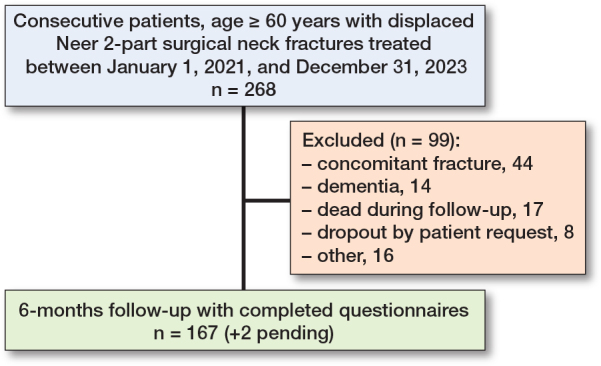
Flowchart of patients treated for Neer 2-part surgical neck fractures. “Other” refers to patients suffering stroke, paralysis of the shoulder not related to the injury, recently operated for unrelated reasons, developmentally disabled, psychotic patients, and terminally ill patients.

This is a prospective cohort study. All patients followed a preregistered protocol [9]. The study design was non-comparative. All patients aged 60 or above treated in a Danish university hospital for a displaced Neer 2-part surgical neck fracture, were considered for inclusion between January 1, 2021, and December 31, 2023 (Figure 3). Discharge data was obtained to ensure completeness.

Reporting followed the STROBE guideline.

### Classifications and outcomes

The first author classified all fractures according to the 16-category Neer [10] and 9-group AO/OTA [11] classifications. As the Neer classification and the AO/OTA are not directly translatable, we decided to classify according to both classification systems [12]. The cohort represents the entire population of patients treated at a Danish university hospital for proximal humerus fractures, and who did not meet the exclusion criteria.

Shoulder function was assessed with the Oxford Shoulder Score (OSS), a patient-administered questionnaire developed to evaluate outcome after elective shoulder surgery [13]. It consists of 12 questions, each answered by a 5-category Likert scale. It focuses on pain and impairment of activities of daily living. The score ranges from 0 to 48, with 48 being the best. It has been validated in non-surgically treated shoulder fractures [14]. Normal values for OSS were adapted from existing data [15].

Health-related quality-of-life was assessed using EuroQol’s 5 dimensions 3 levels (EQ-5D-3L), a generic, patient-administered instrument [16]. It comprises 5 dimensions (mobility, self-care, usual activities, pain/discomfort, anxiety/depression) and a visual analogue scale for the patient’s assessment of overall health. Each dimension has 3 levels of severity. The EQ-5D-3L score ranges from –0.624 to 1.000, with 1 being the best possible score. A negative score represents a condition experienced as “worse than death.” Population norms were adapted. The population norm for Danish females aged 70–79 is 0.82 [17].

### Treatment

The treatment protocol included 4 contacts with healthcare providers. The patient was first seen in the emergency room for pain management and provided with a sling and swathe. The attending doctor conducted a thorough examination, including an assessment of soft tissue and neurovascular status. 2 perpendicular radiographic views were taken to confirm the Neer 2-part surgical neck fracture and to rule out other fractures or dislocations of the shoulder girdle.

Within 2 weeks, the patient was seen in the shoulder fracture clinic. A shoulder specialist informed the patient regarding the injury’s course and nonoperative treatment. Expectations were aligned with pre-injury shoulder function, medical comorbidity, frailty, and patient preferences. Our primary goal when treating older people with shoulder fractures was to restore pain-free function at the shoulder level. The axillary nerve function was assessed by testing muscle contraction and sensory function over the deltoid, with the possibility of a later reverse shoulder arthroplasty in mind. The patient was offered rehabilitation in the municipality from the third week onwards. The exercises’ content, intensity, and duration were decided in collaboration with the physiotherapist. We discouraged the use of slings from this point. All patients were referred to the Fracture Liaison Service for an osteoporosis checkup, including a DEXA scan of the axial skeleton. Patients with fractures that were minimally displaced were subsequently discharged from hospital follow-up.

6 weeks after the injury, patients with Neer 2-part fractures were seen by a shoulder specialist in the shoulder fracture clinic. Progression in rehabilitation was noted, and radiographs were taken to follow radiological healing. Range of motion and rotator cuff integrity were evaluated clinically.

Patients were invited for a clinical examination and evaluation of patient-reported outcome (using EQ-5D-3L and OSS) 6 months after the injury. A final set of radiographs was taken to assess radiological healing.

Operative intervention was considered a failure of non-surgical treatment and recorded as early (0–6 weeks) or late (from 6 weeks). Before the 6-month visit, a nurse not involved in the cohort study collected the completed questionnaires.

Surgery may be considered at any time for patients experiencing persistent or worsening pain beyond the first 4 weeks and who wish to undergo surgery, regardless of radiographic pattern. We used a reverse shoulder arthroplasty with tuberosity osteotomy, fixation to the humeral stem, and rotator cuff reconstruction as the preferred surgical method. This is usually possible within 6 weeks after the trauma.

### Statistics

We used descriptive statistics to present our data, reporting rates, and proportions. Both outcome instruments had a ceiling effect, indicating that the data was skewed toward the higher end. Both median and mean values were calculated. As the median values were slightly higher, we reported the mean and standard deviation to ensure the most cautious estimate. Differences in outcome between the 10-year age bands were tested statistically using the Kruskal–Wallis rank sum test. Outcome data from other cohorts were reported qualitatively, as statistical comparisons of patient outcome could not be performed due to possible confounding.

### Ethics, registration, data sharing plan, use of AI, funding, and disclosures

The protocol was approved by the Scientific Ethics Committee, Region Zealand, Denmark (jr. no. EMN-2021-07413) and the Danish Data Protection Agency, Region Zealand, Denmark (jr. no. EMN-2021-01792). The protocol was registered in “researchregistry6502” [9]. Data is available from the first author upon reasonable request. There has been no use of AI. No external funding has been received. None of the authors has any conflicts of interest. Complete disclosure of interest forms according to ICMJE are available on the article page, doi: 10.2340/17453674.2024.42301

## Results

We treated 268 consecutive patients with displaced Neer 2-part surgical neck fractures between January 1, 2021, and December 31, 2023 (Figures 1–3). The mean age was 76 (SD 8.5). Females accounted for 79%. We excluded 99 patients from the cohort for specified reasons (Figure 3). The remaining 167 patients completed the questionnaires. 8 patients (3%) underwent surgery during the follow-up period, and their outcome was reported separately. The patients who converted to surgery remained in the cohort and delivered their questionnaires 6 months after the date of surgery. 3 patients were identified from the review of discharge diagnosis (Figure 3).

**Figure 4:**
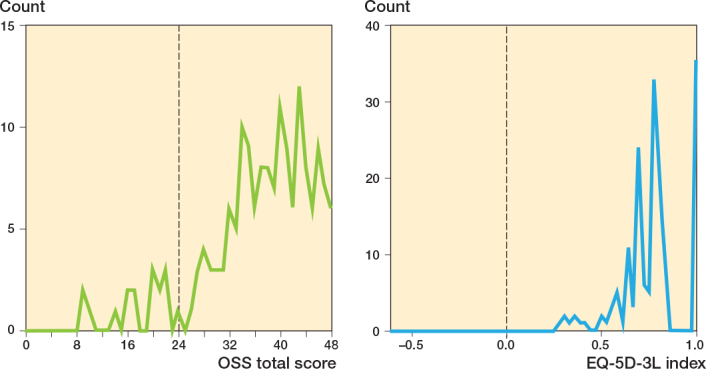
The distribution of OSS and EQ-5D-3L at 6 months. Hatched line is half of full shoulder function (OSS), and quality of life “worse than death” (EQ-5D-3L).

A ceiling effect was identified for both outcome instruments (Figure 4). The mean value for the OSS shoulder score for all age groups was 37.2 (SD 8.1), equivalent to 78% of maximum shoulder function. The expected value in a female population aged 71–80 was 82% [15]. The mean value for EQ-5D-3L was 0.79 (SD 0.16). The population norm for females aged 70–79 was 0.82 [17]. Further improvement in both outcome measures can be expected between 6 and 12 months [8,18]. No significant difference in outcome was found between the age groups for OSS (P = 0.98) and EQ-5D-3L (P = 0.4) (Kruskal–Wallis rank sum test) (Figure 4).

16 of 167 patients (10%) with complete follow-up had an OSS of 24 or below, meaning they had half or less of maximum shoulder function remaining. No patients had an EQ-5D-3L value below 0 (“worse than death”).

8 patients were treated operatively, all with reverse total shoulder arthroplasty, 3 within 6 weeks and 5 later than 6 weeks. The procedures were performed between 3 and 29 weeks. All those operatively treated had severe pain and translated fractures with no bony contact. Interestingly, several others with no bony contact treated nonoperatively regained pain-free shoulder function. The outcome from the surgical group was heterogeneous, with OSS ranging from 10 to 36 and EQ-5D from 0.31 to 0.78. The number did not allow for statistical analysis.

## Discussion

We aimed to evaluate patient-reported outcome in a consecutive series of older people receiving nonoperative treatment for displaced Neer 2-part surgical neck fractures.

We found that, after 6 months, non-surgical treatment in older people with Neer 2-part surgical neck fractures resulted in patient-reported shoulder function and quality of life close to that of the Danish background population. The operation rate was 3%. The population studied is the group most frequently seen in orthopedic units. Following a predefined protocol, we studied a large consecutive cohort of patients in an orthopedic unit. We prioritized reporting the benefits and harms of implementing evidence from randomized trials rather than conducting another randomized trial. It should be mentioned that elements in the rehabilitation protocol were not evidence-based aside from the non-surgical approach [19] and the short period of immobilization [20].

Other prospective cohort studies on Neer 2-part surgical neck fractures have been reported. Court-Brown and McQueen followed 99 patients with varus-impacted 2-part fractures (AO A2.2, mean age 73) for a year and found that nonoperative treatment may increase varus angulation [21]. However, they reported that decreased shoulder function was associated with increasing age but not with increasing varus angulation. All fractures united and 79% of patients obtained good or excellent outcome at 1 year measured with the Neer score (a score rarely used today). This is in correspondence with our results showing values slightly below the level for the background population.

Moreover, they reported 1-year outcome in 126 patients with translated 2-part fractures (AO A3.2, mean age 72). Over half of the patients had more than 66% of displacement on the initial radiographs. They reported that surgery did not improve the outcome, regardless of the degree of translation. They found no correlation between translation or angulation and the ability to return to daily activities. The incidence of non-union was 5% [22]. The patients needing surgery in our cohort may represent this group, as no clinical non-unions were found in our cohort. We cannot rule out some symptomatic non-unions appearing after 6 months.

### Limitations

First, the study was carried out in a single Danish university hospital. Studies of other populations in other places may arrive at different results because of demographic differences and patterns of referral. Second, the results reported in this study apply only to individuals aged 60 or above with displaced Neer 2-part surgical neck fractures, and they cannot be extended to younger patients, high-energy trauma, polytrauma, isolated tuberosity fractures, or fracture-dislocations. Third, the lack of translatability between the Neer and the AO classifications is a challenge for reporting and comparing outcome after surgical neck fractures. Using the 27 subgroup AO form, 6 subgroups (A2.1, A2.2, A2.3, A3.1, A3.2, A3.3) cover most morphologies, including varus and valgus impaction, angulation, translation, and comminution. However, without a definition of displacement, we cannot translate these patterns into Neer 2-part surgical neck fractures [12]. Fourth, observer variation in classifying shoulder fractures is well described [23]. Fifth, it may be argued that patients who underwent late surgery have been disadvantaged. They appear to have lower scores than the average patient. We recognize that the outcomes after surgery seem slightly lower. However, this is not surprising as we operated on the most “severe” cases in terms of patients suffering severe pain. Operating on less severe cases with displaced radiographs may lead to better scores, but it may also result in unnecessary surgery. The discussion on indications and timing of surgery is ongoing. Sixth, it could be objected that performing primary surgery on many more patients could prevent severe pain and late surgery. However, we must consider the high failure rates after locking plate osteosynthesis [24], the unknown revision burden after reverse shoulder arthroplasty, and the cost and opportunity cost associated with increasing surgery.

### Conclusion

Non-surgical treatment in people with Neer 2-part surgical neck fractures aged 60 or above resulted after 6 months in patient-reported shoulder function and quality of life close to that of the Danish background population. The number of clinical failures was low, with less than 3% needing surgery.

## Notes

SB wrote the protocol. Data were collected by SB, SAB, and LLH. Data analysis was performed by SAB. SB wrote the manuscript. All authors contributed to the revision of the manuscript.

The authors would like to thank Maria D. Nielsen and Birgitte Bonkegaard for maintaining the cohort and ensuring high completeness.

Handling co-editor: Taco Gosens

*Acta* thanks Roger John Emery and Antti P Launonen for help with peer review of this manuscript.
